# Correlates of Primary Healthcare Access and the Moderating Effects of Gender: A Cross Sectional Analysis of Irish Migrants

**DOI:** 10.1007/s10903-021-01208-5

**Published:** 2021-05-17

**Authors:** Jeff Moore

**Affiliations:** School of Human Development, Dublin City University, Dublin, Ireland

**Keywords:** Irish migrants, Healthcare access, Social support

## Abstract

Despite long established comparatively poor health outcomes there has been limited research into the healthcare access of Irish migrants in the UK. This study examines the relationship between demography, self-reported health (SRH) and social support and healthcare access and the influence of gender on these associations. Data was collected as part of a community-based action research project with Irish migrants in London (*n* = 790). Hierarchical logistic regression was used to predict self-reported access to a GP (compared with no reported access). The effect of gender was measured via interactions entered in the second step of the model. Older participants and males were less likely to report GP access. SRH was a significant predictor. Gender moderated the relationship between SRH, social support, employment and GP access. Findings highlight the help-seeking vulnerability of male and older Irish migrants and the potential of social support in promoting healthcare access for males.

## Introduction

In most healthcare systems, black and minority ethnic (BME) populations have experienced poor access and barriers to certain services. Some studies show that the association between ethnicity and health care use can be partly explained by education, knowledge and language proficiencies [1]. However, studies of healthcare access for BME communities have commonly compared access with the general population and we know less about the within group factors that predict formal health care access for migrants.

A sizable volume of research shows that Irish people in the UK experience poor health when compared to the general population [2]. Despite this elevated risk, there is limited information on the health service access of this community. Nazroo et al. found that Irish people were not less likely to access a GP when compared to the general UK population [3]. However, qualitative research indicates some older Irish migrants experience cultural insensitivity in the UK healthcare system and may be reluctant to seek care in formal setting [4].

Women in the UK generally make more GP consultations [5]. Review studies indicate that traditional male traits of stoicism and control inhibit health related help-seeking [5]. Studies on migrant healthcare access rarely include analysis of gender and those that do show mixed results [6]. In the context of Irish migrants, studies indicate that older male migrants are more vulnerable to social isolation and substances misuse, which may hinder healthcare access [4]. Irish female migrants have traditionally demonstrated higher levels of economic activity with many involved in teaching and nursing roles in the UK [7] which may play a role in promoting health care access. However, little research has specifically examined the influence of gender in health care access for this community.

## The Present Study

Our primary aims is to explore the socio-demographic, social support (SS) and health related factors that predict self-reported healthcare access. In addition, we examine the moderating effect of gender on the relationship between predictor variables and healthcare access.

## Methods

### Participants

Data were collected in 2011 as part of a community-based action research project with first and second-generation Irish people in London (*n* = 790). Table 1 provides sample characteristics.

**Table 1:** Sample characteristics

	*N*	%	*M*
Gender			
Male	306	39.95	
Female	460	60.05	
Country of birth			
Ireland	572	73.90	
UK	202	26.10	
Age			
18–29.00	205	26.49	
30.00–41.00	186	24.03	
42.00–64.00	191	24.68	
65.00+	192	24.81	
SES			
Higher managerial, administrative, professional	37	8.10	
Intermediate managerial, administrative, professional	223	48.80	
Supervisory, clerical, junior managerial	137	29.98	
Skilled manual workers	22	4.81	
Semi-skilled and unskilled manual workers	38	8.32	
SS score (OSSS-III)			9.25
SS categories			
Poor social support	306	43.53	
Moderate social support	311	44.24	
Strong social support	86	12.23	
SRH			
Very poor	7	0.97	
Poor	59	8.16	
Fair	120	16.60	
Good	271	37.48	
Very good	266	36.79	
Self-reported GP access	509	79.12	

### Data Collection

The study employed a non-proportionate purposeful sampling strategy targeting Irish people in London via Irish networks. Data were collected via an on-line survey and in community based organisations (CBOs) across London. Previous studies have detailed similarities between this sample and the Irish community in London as per the census population [8]. All participants provided informed consent at the outset of the survey. Ethical approval was obtained from Middlesex University Research Ethics Committee prior to commencement of data collection.

## Measures

### Healthcare Access

Healthcare access was assessed using a single-item self-report measure (poor to very good). Participants were asked to specify where they go for advice for health-related difficulties. Respondents were provided with a list which included general practitioners (GP), CBOs, friends and family and the internet.

### Predictors Variables

General health was measured using a single-item measure of self-rated health (SRH). Participants were asked to disclose number of ailments. SS was measured using the Oslo 3-item social support scale (OSSS-3). The OSSS-3 has been widely used and studies have evidenced the scales feasibility and predictability with respect to psychological distress [9]. Cronbach’s alpha in the current study was acceptable (ɑ = 0.58). Information on socio economic status (SES) was collected using the office of national statistics UK SOC2010 unit groups.

### Data Analysis

A hierarchical logistic regression was conducted to test associations with health and social related variables and self-reported healthcare access. The dependent binary variable was access to GP for health concerns/not accessing a GP for health concerns. Moderation effects were evaluated by using the baseline model, at step one, to which interaction terms were added between gender and demographics (age, SES and country of birth), gender and SS and gender and health related variables at the second step. The assumption of linearity, normality and homoscedasticity were met. All predictor variables were centred.

## Results

**Fig. 1:**
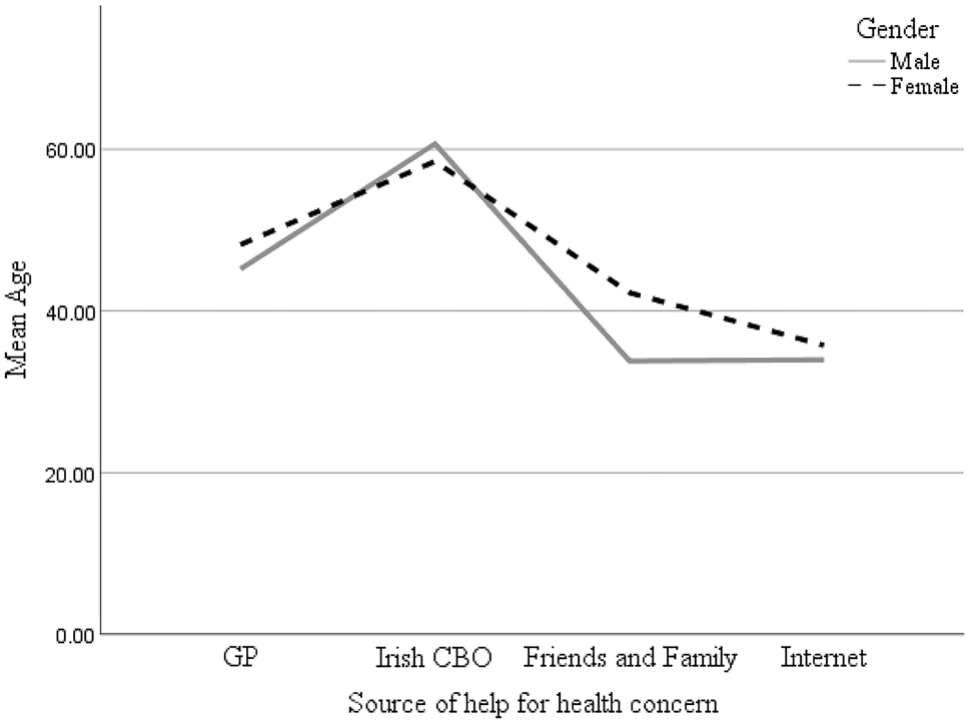
Mean age by source of help for health concerns

Bivariate correlation analysis did not find high inter correlations between predictor values. The strongest correlation was between age and number of ailments (*R* = 0.55, *p* = 0.00, full results available on request). Table 2 shows the regression results. Model one was a significant predictor of health care access, χ^2^ (7) = 20.81, *p* = 0.004. In the first step, men were 1.80 times less likely to report seeking help via a GP for a health concern. SRH was significantly associated with seeking help. Which each year aged participants were 1.04 times less likely to seek help from a GP with health concerns. Figure 1 presents mean age per source of health care seeking, showing that those who sought help from Irish CBOs have the oldest age profile.

**Table 2:** Regression table predicting self-reported healthcare access

	*B*	*S.E.*	*P*	*OR*	*Lower*	*Upper*	*B*	*S.E.*	*P*	*OR*	Lower	Upper
Model 1												
Age	− 0.03	0.014	0.020	0.969	0.944	0.995	− 0.033	0.015	0.023	0.967	0.940	0.995
Country of birth (Ireland/UK)	0.041	0.325	0.901	1.041	0.551	1.969	0.065	0.334	0.847	1.067	0.554	2.055
Gender (male/female)	− 0.592	0.273	0.030	0.553	0.324	0.945	− 0.855	0.488	0.079	0.425	0.163	1.105
SES	− 0.041	0.441	0.926	0.960	0.404	2.279	0.346	0.468	0.460	1.413	0.564	3.538
SRH	− 0.429	0.232	0.064	0.651	0.413	1.025	− 0.69	0.278	0.013	0.501	0.291	0.865
SS Scale (OSSS-3)	0.053	0.066	0.426	1.054	0.926	1.201	0.036	0.070	0.604	1.037	0.904	1.189
Number ailments	0.063	0.223	0.779	1.065	0.688	1.648	− 0.075	0.236	0.750	0.927	0.584	1.473
Model 2												
Gender × SRH							1.014	0.520	0.051	2.757	0.995	7.637
Gender × SES							− 2.230	0.927	0.016	0.108	0.017	0.662
Gender × country of birth							0.056	0.676	0.934	1.057	0.281	3.976
Gender × age							0.026	0.028	0.352	1.027	0.971	1.085
Gender × SS							0.292	0.138	0.035	1.340	1.021	1.757
Gender by ailments							0.935	0.473	0.048	2.547	1.008	6.435
Model fit.												
* −2LL*	361.72						345.72					
* R*^2^	0.085						0.147

**Fig. 2:**
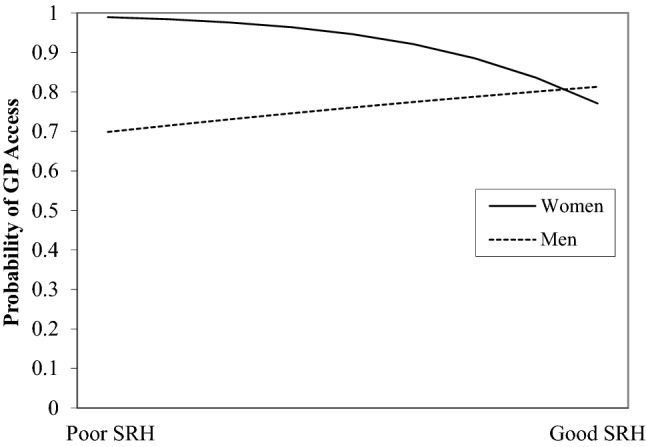
Plotting the probability of self-reported access to GP by SRH and gender (adjusted for covariates)

**Fig. 3:**
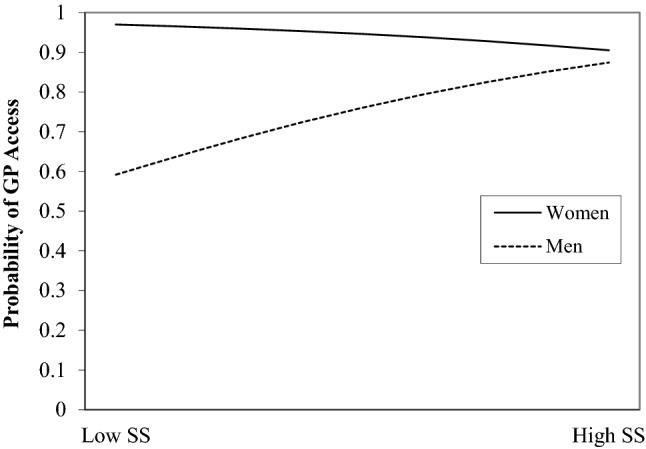
Plotting the probability of self-reported access to GP by SS and gender (adjusted for covariates)

With the inclusion of interaction terms, model 2 was a significant predictor, χ^2^ (13) = 36.81, *p* = 0.01. Gender significantly moderated the relationship between SRH and help-seeking. As illustrated by Fig. 2, for men the likehood of reporting GP access decreased as SRH gets worse whereas for women as SRH worsens the likehood of GP access increases. Results were similar for number of ailments. Gender moderated the effect of SS on GP access (Fig. 3). For women help seeking remained stable irrespective of SS, whereas for males increased SS was associated with higher likelihood of reporting GP access. Finally, there was no difference in GP access by gender for those with low SES. However, higher SES resulted in an increased likelihood of GP access for females but a reduced likelihood for males.

## Discussion

Contrary to what we might expect considering the health profile of older Irish migrants [4], increased age was not associated with increased likehood of healthcare access. Previous studies have demonstrated that older vulnerable Irish migrants show a higher degree of reliance on Irish community organisation and this be a factor in lower levels of GP access [8]. Others have pointed to a health stoicism as a cultural component in older Irish communities and this may account for this finding [10].

Results indicate that Irish male migrants are less likely to report healthcare seeking when they perceive their health to be poor. This may be related to traditional masculine attitudes to help seeking (for a detailed review [5]). A preference for self-management, self-medication or fear of diagnoses may also be factors. In contrast, females are often better able to interpret symptoms that may increase health care access in poor health [5]. Our results support previous research which demonstrates that SS, such as the influence of female partners, is an important driver in healthcare access of males [5] whereas female access is not influenced by SS.

The study engaged one of the largest specific samples of Irish people in the UK; however, the sampling strategies means the study is non-representative. The study employed commonly used and valid measures of health and social support. The question on GP access focused on seeking advice rather than attendance for specific symptoms and this may have effected male responses, as studies demonstrate that men are more likely to seek help for physical symptom [5]. As the study was cross sectional, we cannot confirm the direction of associations and are not able to rule out reserve causality.

## New Contribution to the Literature

This report provides the first specific analysis of primary care access and the moderating effect of gender for Irish migrants in the UK. Our results support long held assumptions on the help-seeking vulnerability of older and male migrants. The study indicates that SS may be a particularly important promotive factor in the health related help seeking of Irish male migrants.
